# Dietary determinants of aflatoxin B_1_-lysine adduct in pregnant women consuming a rice-dominated diet in Nepal

**DOI:** 10.1038/s41430-019-0554-2

**Published:** 2020-01-02

**Authors:** Johanna Y. Andrews-Trevino, Patrick Webb, Gerald Shively, Beatrice Rogers, Kedar Baral, Dale Davis, Krishna Paudel, Ashish Pokharel, Robin Shrestha, Jia-Sheng Wang, Kathy S. Xue, Shibani Ghosh

**Affiliations:** 1 Friedman School of Nutrition Science and Policy, Tufts University, 150 Harrison Avenue, Boston, MA 02111 USA; 2 Department of Agricultural Economics, Purdue University, 403 West State Street, West Lafayette, IN 47907 USA; 3 Department of Community Health Sciences, Patan Academy of Health Sciences, Lalitpur, Nepal; 4 Helen Keller International, P.O. Box, 3752 Kathmandu, Nepal; 5 Kanti Children’s Hospital, Kathmandu, Nepal; 6 University of Georgia, 206 A Environmental Health Science Building, Athens, GA 30602 USA

**Keywords:** Biomarkers, Risk factors

## Abstract

**Background:**

Aflatoxins are found in diverse foods widely consumed worldwide. This study investigated the association between aflatoxin exposure and (a) consumption of specific foods, (b) dietary diversity (DD), and (c) seasonality.

**Methods:**

Women enrolled in the AflaCohort Study in Banke, Nepal (*n* = 1648) were asked how often they ate certain food items in the past 7 days and 24 h. Serum aflatoxin B_1_-lysine (AFB_1_-lys) adduct levels, measured during pregnancy, were determined using high-performance liquid chromatography. Multivariable ordinary least squares and quantile regression models were used to examine incremental increases in AFB_1_-lys adduct levels per frequency of food consumption and the relationship between DD, seasonality, and increases in AFB_1_-lys adduct.

**Results:**

Roughly 94% of women were exposed to aflatoxin (geometric mean 1.37 pg/mg). Women in the 30th, 50th, and 70th quantiles of aflatoxin exposure who reported one more occasion of maize consumption in the past week showed increases in AFB_1_-lys adduct levels: 0.094, 0.112, and 0.109 pg/mg (*p* < 0.05, all). Women in the 30th, 50th, 70th, and 90th quantiles of exposure who reported one more occasion of groundnut consumption in the past week also showed increases in AFB_1_-lys adduct levels: 0.058 (*p* < 0.001), 0.085 (*p* < 0.01), 0.133 (*p* < 0.001), and 0.133 (*p* < 0.001) pg/mg. Winter month recruitment was positively associated with AFB_1_-lys adduct levels at all quantiles of aflatoxin exposure (range: 0.313–1.101 pg/mg, *p* < 0.001). DD was not predictive of aflatoxin exposure.

**Conclusions:**

Our findings justify integrated approaches to aflatoxin reduction, including regulatory, agricultural, and food safety interventions across the value chain and at the household level.

## Introduction

In South Asia, women and young children are at risk of exposure to aflatoxin, a naturally occurring toxin produced by *Aspergillus* fungi [1, 2]. Acute aflatoxicosis can cause coma or death. Chronic, low level exposure to aflatoxin is harmful to human health [3]. Evidence shows placental transfer of aflatoxin from mother to fetus [4] and linkages to impaired linear growth in childhood [4, 5].

Exposure occurs primarily through the consumption of contaminated foods. Maize, chilies, spices, oilseeds, and nuts are especially susceptible to aflatoxin contamination [6–8]. When ruminants ingest feed contaminated with aflatoxin they metabolize and excrete the metabolite, aflatoxin M_1_ (AFM_1_), in milk [9]. Aflatoxins are difficult to detect and remove because they are unobservable to the consumer and relatively resistant to thermal inactivation [10, 11].

Populations at particularly high risk of chronic aflatoxin exposure are resource-scarce, have limited dietary variety, store foods for long periods, and rely on highly susceptible foods including maize and groundnuts [12, 13]. Access to improved dietary diversity (DD) may lower aflatoxin exposure by lessening the dependence on aflatoxin-prone foods and counteracting the toxicity [14, 15], particularly in those consuming monotonous diets [16, 17].

This study was conducted to determine: (a) if the frequency of consumption of susceptible agricultural commodities was associated with aflatoxin exposure in pregnancy in Nepali women, (b) if increased DD was associated with lower levels of aflatoxin exposure, and (c) whether aflatoxin exposure levels vary seasonally.

## Methods

### Study population

The AflaCohort Birth Cohort Study (2015–2019) was conducted in Banke, a tropical district (Province 5) in the southern plains of Nepal. A rolling recruitment strategy was used to enroll 1675 healthy pregnant women. The sample size was calculated assuming an alpha of 0.05, power of 80%, attrition of 20%, and design effect of 1.5. This allowed the detection of a −0.207 standard deviations (SD) difference in postnatal height-for-age *Z*-score for every 1-unit increase in log average maternal AFB_1_-lys adducts.

Eligibility criteria included: <30 weeks pregnant, age 16–49, singleton pregnancy, living, and planning to give birth in the study area. This analysis used data collected during pregnancy (July 2015–August 2016); nation-wide strikes interrupted data collection for 3 months and resumed in December 2015.

The women (or their legal guardians) gave verbal and written consent prior to participation. The Nepal Health Research Council (295/2014), and the Tufts Institutional Review Board (11535) approved this study.

### Data collection

Trained interviewers administered electronic surveys. Surveys included a single qualitative 7 and 24 h food frequency questionnaire (FFQ) [18] to determine the frequency of consumption of 49 predetermined food items. The food items included were based on previous dietary assessments in this population [19]. Consumption data were also collected for the past year.

Upon survey completion, interviewers measured height, weight and mid-upper arm circumference (MUAC) to the nearest 0.1 cm and 0.1 kg using ShorrBoard^®^ Measuring Boards, 874 Seca Scales, and 65 cm adult measuring tapes, respectively.

Within a week of survey completion, nurses visited the women and collected a 3–5 mL antecubital vein blood sample. Blood samples were transported on wet ice to a local laboratory for processing. Samples were air-shipped on wet ice to the Patan Academy of Health Sciences to be stored at −80 °C until they were ready to be air-shipped on dry ice to the Wang laboratory at the University of Georgia.

### Data analysis

A total of 1650 gestational serum samples were analyzed for AFB_1_-lys adducts, an established biomarker of dietary aflatoxin exposure over the previous 2–3 months [20]. The levels of AFB_1_-lys adducts were measured using a validated high-performance liquid chromatography (HPLC) with fluorescence detection method [21].

After deactivation in 56 °C water bath for 30 min, ~150 μL of each sample were digested by pronase (pronase: total protein, 1:4, w/w) at 37 °C for 3 h to release adducts. The digests were extracted and purified by passing through a Waters MAX SPE cartridge, eluted with 2% formic acid in methanol, vacuum-dried with a Labconco Centrivap concentrator (Kansas City, MO), and reconstituted with 25% methanol water for HPLC-fluorescence detection.

An Agilent 1200 HPLC-fluorescence system (Santa Clara, CA) was used to quantify AFB_1_-lys adducts. The mobile phases consisted of buffer A (20 mM NH_4_H_2_PO_4_, pH 7.2) and buffer B (100% Methanol), running at a gradient to allow separation within 25 min of injection, with a typical retention time for AFB_1_-lys adduct at ~13 min. Separation was achieved using Zorbax Eclipse XDB-C18 reverse phase column (5 micron, 4.6 × 250 mm) equipped with a guard column, maintained at 25 °C and a flow rate of 1 mL/min during analysis. Sample injection volume was 100 μL. Excitation and emission wavelengths for detection were 405 and 470 nm, respectively. Calibration curves of authentic standard were generated weekly. Quality assurance and quality control procedures included simultaneous analysis of one authentic standard for every ten samples, and two daily quality control samples. The average recovery rate was 90% for the report, the AFB_1_-lys concentration was adjusted by albumin concentration, measured via UV/Visible spectrophotometry. Samples below the limit of detection (LOD) (0.4 pg AFB1-lysine/mg albumin) were substituted with a constant value of half the LOD for statistical analysis [22].

Minimum DD scores were computed using Minimum Dietary Diversity for Women of Reproductive Age (MDD-W) guidelines [23]. Food items from the 24-h FFQ were categorized into one of ten food groups: [1] grains/roots/tubers, [2] pulses, [3] nuts and seeds, [4] dairy, [5] meats, [6] eggs, [7] dark green leafy vegetables (DGLV), [8] other vitamin A sources, [9] other vegetables, [10] other fruits. A dichotomous MDD indicator was created to calculate whether women achieved MDD (consuming ≥ 5 of the 10 food groups in the previous 24 h).

Covariates analyzed included age, education, wealth status, MUAC, season, and Village Development Committee with those included in the models being selected based on their potential for confounding.

Seasonal variations in aflatoxin exposure were examined for autumn, prewinter, winter, spring, summer, and monsoon seasons. Autumn is characterized by wet, cool weather, while the prewinter and winter are cooler and drier [24]. Spring and summer are warm and dry. The summer months are the hottest while the rainy/monsoons are the most humid. Annual rainfall during the survey period (mean = 1232 mm, SD = 577) did not differ significantly (*t* = 0.12) from prior years—January 1999–December 2014 (mean = 1263, SD = 468) with no differences in monthly averages between the two periods [25].

Principal component analysis [26–29] was used to construct a composite measure for household wealth. Data on type of roof, floor, walls, toilet, cooking fuel, piped water, number of household members, and asset ownership (livestock, radio, television, mobile phone, bicycle, motorcycle, electric fan) were used to construct the variable.

Data were divided into quintiles of aflatoxin B_1_-lys adducts (lowest: ≤0.5823; low: >0.5825 to ≤0.9219; middle: >0.9219 to ≤1.4322; high: >1.422 to ≤2.9315; very high: >2.9315 pg/mg). Nonnormally distributed AFB_1_-lys data were natural log-transformed for all statistical analyses.

Two-sided Student’s *t* tests and analysis of variance and chi-squared tests tested continuous variables and dichotomous variables, respectively. Covariate-adjusted parameter estimates with 95% confidence intervals were computed using ordinary least squares (OLS) and quantile regression (QR) [30]. QR models were used to quantify the associations of aflatoxin-prone-food consumption frequency and maternal DD at different points of the aflatoxin distribution (10th, 30th, 50th, 70th, and 90th quantiles). Restricted cubic splines and nonparametric smoothing curves helped test for an unadjusted nonlinear relationships [31]. Variance inflation factors helped diagnose multicollinearity among the predictor variables in the regression models. Significance levels were set at *p* < 0.05. Statistical analyses were conducted using Stata 14.2 (StataCorp LP).

## Results

**Fig. 1:**
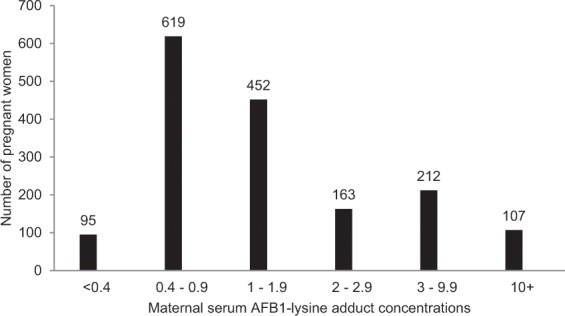
AFB_1_-lysine adducts in serum of pregnant women.

AFB_1_-lys adducts (range: 0.4–147 pg/mg albumin) were found in 94% of samples (Fig. 1; mean concentration 3.2 ± 8.3 pg/mg albumin, geometric mean 1.37 pg/mg albumin, CI: 1.3–1.4).

**Fig. 2:**
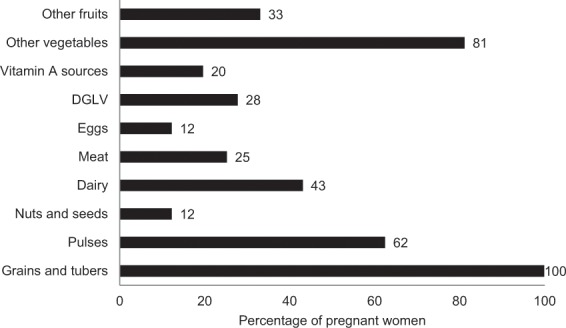
Percent of pregnant women consuming foods from various food groups in the past 24 h.

In the past 24 h, 100% women consumed rice, 62% consumed pulses, while 12% consumed nuts/seeds (Fig. 2). Dairy was more commonly consumed than meat (43% versus 25%) in the past 24 h. While over 80% reported consuming vegetables, the reported 24-h consumption of fruit, eggs, DGLV, and vitamin A-rich sources was low (33%, 12%, 28%, and 20%, respectively). Only 39% achieved MDD.

Age and MUAC were significantly negatively associated with maternal AFB_1_-lys adduct concentrations in bivariate analyses. Hemoglobin level and winter season were significantly positively associated with aflatoxin exposure (Table 1).

**Table 1:** Serum aflatoxin B_1_-lysine adduct levels by sociodemographic and health characteristics of pregnant Nepalese women enrolled in the AflaCohort Study^a^.

	*n*	*n* %	Mean AFB_1_	SD	Geo mean AFB_1_	95% CI	Highest AFB_1_ quintile^b^	
							*n*	%
Age category						**		
<20	347	21.1	4.4	11.2	1.6	1.4–1.9	77	22.2
21–24	627	38.1	3.3	7.6	1.4	1.3–1.5	117	18.7
25–29	470	28.5	2.4	4.4	1.2	1.1–1.4	93	19.8
30–34	135	8.2	3.0	12.9	1.2	1.0–1.4	26	19.3
35+	69	4.2	2.2	2.6	1.3	1.0–1.6	16	23.2
Schooling								
None	606	36.8	2.5	5.1	1.3	1.2–1.4	121	19.9
Some primary (1–5)	321	19.5	3.1	5.6	1.0	1.3–1.6	68	21.2
Some secondary (6–10)	577	35.0	4.2	12.0	1.4	1.3–1.6	117	20.3
More than secondary (10+)	144	8.7	2.6	5.1	1.3	1.1–1.5	23	16.0
Wealth Index Quintile								
Poorest	328	19.9	3.5	9.3	1.4	1.3–1.6	73	22.3
Poor	331	20.1	2.8	6.4	1.2	1.1–1.4	60	18.1
Middle	329	20.0	2.7	6.4	1.3	1.1–1.4	55	16.7
Rich	328	19.9	4.0	9.4	1.5	1.4–1.8	80	24.4
Richest	332	20.2	3.1	9.3	1.4	1.2–1.5	61	18.4
Religion								
Hindu	1258	76.3	3.2	8.5	1.3	1.3–1.4	244	19.4
Buddhist	5	0.3	3.4	5.3	1.2	0.0–9.3	1	20.0
Muslim	362	22.0	3.1	7.2	1.4	1.2–1.6	78	21.6
Christian	23	1.4	5.4	11.2	1.8	1.0–3.2	6	26.1
Ethnicity								
Brahmin	77	4.7	3.5	9.1	1.5	1.2–2.0	21	27.7
Chettri	299	18.1	3.7	9.2	1.4	1.3–1.6	59	19.7
Tharu	168	10.2	2.6	7.1	1.0	0.8–1.2	27	16.1
Muslim	359	21.8	3.0	7.1	1.4	1.3–1.6	76	21.2
Dalit	380	23.1	2.9	8.6	1.3	1.2–1.5	73	19.2
Other	365	22.2	3.5	8.5	1.5	1.4–1.7	73	20.0
Anemia (hemoglobin < 11 g/dL)^c^								
No	976	59.3	3.5	9.4	1.4	1.3–1.5	207	21.2
Yes	670	40.7	2.7	6.4	1.3	1.2–1.4	121	18.1
Maternal stature								
Short/average (>145 cm)	1422	86.4	3.2	8.3	1.4	1.3–1.4	284	20.0
Very short (≤145 cm)	224	13.6	3.2	8.3	1.5	1.3–1.7	45	20.1
MUAC^d^								
Average (>23 cm)	1099	66.7	3.2	8.5	1.4	1.3–1.4	216	19.7
Low (≤23 cm)	549	33.3	3.3	7.9	1.4	1.3–1.5	113	20.6
Minimum Dietary Diversity^e^								
No	1003	60.9	3.0	7.3	1.3	1.2–1.4	200	19.9
Yes	645	39.1	3.6	9.6	1.4	1.3–1.6	129	20.0
Season of measurement						***		
Spring	514	31.2	2.2	5.1	1.2	1.1–1.3	73	14.2
Summer	391	23.7	1.3	2.8	0.8	0.8–0.9	31	7.9
Rainy/Monsoon	32	1.9	1.2	1.3	0.8	0.6–1.1	2	6.3
Autumn	0	0	n/a	n/a	n/a	n/a	n/a	n/a
Prewinter	238	14.4	6.6	14.1	2.6	2.2–3.1	92	38.7
Winter	473	28.7	4.2	9.6	1.8	1.6–2.0	131	27.7

*Geo* geometric, *MDD-W* minimum dietary diversity score for women, *MUAC* mid-upper arm circumference

**p < 0.01; ***p < 0.001

^a^Numbers do not always add up due to missing responses

^b^Highest quintile > 2.9 pg/mg. AFB_1_ values were log-transformed before analysis

^c^Mean hemoglobin of 11.2 ± 1.2 g/dL

^d^Mean MUAC of 24.1 ± 2.5 cm

^e^Food and Agriculture Organization minimum dietary diversity defined as consuming ≥5 of the ten food groups in the previous 24 h, mean dietary diversity score of 4.2 ± 1.5

Weekly consumption frequencies included 31% reporting consumption of groundnuts, 3% reporting maize, and 2% reporting both groundnuts and maize (Table 2). The weekly mean frequency of maize and groundnuts consumption was 2.4 ± 2.2 and 2.8 ± 2.2, respectively. AFB_1_-lys adduct levels were significantly higher in maize and groundnut consumers (7.9 pg/mg albumin adducts) compared with those who did not eat maize or groundnuts (2.4 pg/mg albumin adducts, *p* < 0.0001) in the past week.

**Table 2:** Maize, groundnut, and chili consumption in the past week and year^a^.

	*n*	% or mean ± SD	*p* value log AFB_1_	Mean AFB_1_	SD	Geo mean AFB_1_	95% CI	*p* value log AFB_1_	Highest AFB_1_ quintile^b^	
									*n*	%
Maize and/or groundnut consumption										
Consumption in past week										
None (Ref)	1050	63.7		2.4	6.0	1.1	1.1–1.2		156	14.9
Maize only	49	3.0		5.7	13.3	1.8	1.2–2.6	**	14	28.6
Groundnuts only	518	31.4		4.4	10.6	1.8	1.7–2.0	***	147	28.4
Both	31	1.9		7.9	13.3	3.2	2.0–5.1	***	12	38.7
Maize										
Consumption in past week										
No	1568	95.1		3	7.9	1.3	1.3–1.4		303	19.3
Yes	80	4.9		6.5	13.3	2.2	1.7–3.0	***	26	32.5
Frequency of consumption (times/week)		2.4 ± 2.2	***							
Consumption in past year										
No	282	17.2		3.2	7.4	1.5	1.3–1.7		62	22.0
Yes	1362	82.9		3.2	8.5	1.4	1.3–1.4		267	19.6
Groundnut										
Consumption in past week										
No	1099	66.7		2.5	6.6	1.2	1.1–1.2		170	15.5
Yes	549	33.3		4.5	10.8	1.9	1.7–2.1	***	159	29.0
Frequency of consumption (times/week)		2.8 ± 2.2	***							
Consumption in past year										
No	45	2.7		2.3	4.0	1.2	0.9–1.7		6	13.3
Yes	1601	97.3		3.2	8.4	1.4	1.3–1.5		323	20.2
Chili^c^										
Consumption in past year										
No	4	0.2		0.7	0.3	0.6	0.3–1.3		0	0.0
Yes	1642	99.8		3.2	8.3	1.4	1.3–1.4		329	20.0
Milk										
Consumption in past week										
No	983	59.7		3.3	8.9	1.4	1.3–1.4		189	19.2
Yes	665	40.4		3	7.2	1.4	1.3–1.5		21	20.0
Frequency of consumption (times/week)		5.5 ± 4.0								
Consumption in past year										
No	117	7.1		2.2	3.5	1.1	0.9–1.3		19	16.2
Yes	1531	92.9		3.3	8.5	1.4	1.1–1.5*		310	20.3

*AFB1* aflatoxin B1, *SD* standard deviation, *Geo* geometric, *CI* confidence interval, *Ref* reference category

*p < 0.05; **p < 0.01; ***p < 0.001

^a^Numbers do not always add up due to missing responses

^b^Highest quintile > 2.9 pg/mg. AFB_1_ (pg/mg) values were log-transformed before analyses

^c^Data on weekly consumption of chilies were not available

Weekly maize consumption did not vary across wealth quintiles and was positively associated with maternal education (*p* < 0.01) (Table 3). Weekly groundnut consumption, in contrast, was positively associated with wealth status (*p* < 0.01) but not with maternal education. Frequency of maize (*p* < 0.05) and groundnut (*p* < 0.001) consumption in the past week were significantly higher for women recruited in the winter.

**Table 3:** Average frequencies of maize and groundnut consumption in the previous week by education, wealth, and season of measurement.

	*n*	%	Maize		Groundnut	
			Frequency	SD	Frequency	SD
Schooling						
None	606	36.8	0.1	0.5**	0.9	1.8
Some primary (1–5)	321	19.5	0.1	0.6	1.0	2.0
Some secondary (6–10)	577	35.0	0.2	1.0	0.8	1.8
More than secondary (10+)	144	8.7	0.1	0.7	0.9	1.7
Wealth Index						
Poorest	328	19.9	0.1	0.4	0.7	1.4**
Poor	331	20.1	0.1	0.8	0.8	1.8
Middle	329	20.0	0.1	0.5	1.1	2.0
Rich	328	20.0	0.1	0.9	1.0	1.7
Richest	332	20.1	0.2	0.9	1.1	2.0
Season						
Nonwinter	937	56.9	0.1	0.6**	0.4	1.1***
Winter	711	43.1	0.2	1.0	1.6	2.3

*SD* standard deviation

**p < 0.01; ***p < 0.001

Annual consumption frequencies included 83% reporting maize, 97% groundnut, 100% chilies, and 93% reporting consuming milk (Table 2). No association was detected between aflatoxin exposure and annual consumption of maize or groundnuts. Low variability in annual chili consumption limited our ability to test the association with aflatoxin exposure. Annual milk consumption was positively associated with AFB_1_-lys adduct concentrations (*p* < 0.05). Neither annual wheat nor rice consumption was associated with maternal aflatoxin exposure (data not shown).

In the adjusted OLS model, groundnut consumption in the past week (0.730, *p* < 0.001) and the winter season (2.339, *p* < 0.001) were significant predictors of maternal AFB_1_-lys adduct levels (Table 4). In the QR models, maize and groundnut consumption were heterogeneously positively associated with higher aflatoxin. Every additional occasion of reported weekly maize consumption was associated with higher AFB_1_-lys adduct concentrations in the 30th (0.094, *p* < 0.05), 50th (0.112, *p* < 0.05), and 70th quantiles (0.109, *p* < 0.05) of exposure. In contrast, reported weekly maize consumption was not associated with aflatoxin exposure in the OLS regression or in the QR model in the 10th and 90th quantiles of exposure.

**Table 4:** Multivariate ordinary least squares and quantile regression analysis of the association between weekly maize and groundnut consumption and maternal serum aflatoxin B_1_-lysine adduct levels.

	OLS	Q10	Q30	Q50	Q70	Q90
Maize consumption^b^	0.549 (0.281)	0.091 (0.054)	0.094 (0.041)*	0.112 (0.051)*	0.109 (0.048)*	0.147 (0.111)
Groundnut consumption^b^	0.730 (0.121)***	0.037 (0.027)	0.058 (0.016)***	0.085 (0.026)**	0.133 (0.026)***	0.133 (0.030)***
Milk consumption^c^	0.906 (0.799)	0.630 (0.221)**	0.194 (0.108)	0.230 (0.106)*	0.173 (0.128)	0.066 (0.244)
Dietary diversity score	−0.229 (0.149)	0.064 (0.029)*	0.004 (0.020)	0.008 (0.018)	−0.012 (0.026)	−0.057 (0.053)
Winter season	2.339 (0.430)***	0.313 (0.091)**	0.460 (0.059)***	0.552 (0.066)***	0.623 (0.085)***	1.101 (0.130)***
Model Adjusted *R*^2^	0.0639	0.0539	0.0698	0.0801	0.1010	0.1367

Standard errors in parentheses; *n* = 1648

*MUAC* mid-upper arm circumference, *OLS* ordinary least squares, *Q* quantile

^a^OLS regression

^b^Number of times in past week

^c^Consumed in past year (yes/no)

*p < 0.05; **p < 0.01; ***p < 0.001. Models adjusted for age, education, MUAC, wealth index and Village Development Committee (VDC)

Women in the 30th, 50th, and 70th quantiles of exposure who reported one more occasion of weekly groundnut consumption experienced significantly higher aflatoxin levels: 0.058 (*p* < 0.001), 0.085 (*p* < 0.01), and 0.133 (*p* < 0.001) pg AFB_1_-lys adducts per mg of albumin. Similarly, women in the 90th quantile of exposure reporting one more occasion of weekly groundnut consumption showed significantly higher concentrations of AFB_1_-lys adduct (0.133, *p* < 0.001). However, weekly groundnut consumption was not associated with aflatoxin for women in the 10th quantile of exposure; this may be a function of lower groundnut consumption in women with the lowest aflatoxin levels. Restricted cubic spline analyses found no evidence of a threshold effect between either weekly maize or groundnut consumption and exposure. This suggests that a linear relationship hypothesis between weekly consumption and exposure cannot be rejected for this sample, i.e., frequent consumption results in higher values in blood.

Women in the 10th and 50th quantiles of exposure who reported milk consumption had higher aflatoxin exposure (0.63 (*p* < 0.01) and 0.23 (*p* < 0.05), respectively) than those who did not consume milk in the past year. DD was not associated with maternal aflatoxin exposure in the OLS or at most quantiles in the QR models. DD scores were significantly positively associated with maternal aflatoxin in the 10th quantile of exposure (0.064, *p* < 0.05). The association between winter season and AFB_1_-lys adduct concentration was positive across all quantiles.

## Discussion

Biomarker data show that the majority of the women were exposed to aflatoxin during pregnancy. Diet-associated aflatoxin exposure in these women seems to be driven by groundnut and maize consumption and is highly variable by season of measurement. Contrary to expectations, results showed no association between DD and maternal aflatoxin levels.

The geometric mean of serum maternal AFB1-lysine adduct concentration of 1.37 pg/mg albumin (95% CI: 1.30, 1.44 pg/mg albumin) in this cohort was lower than average concentrations found in similar studies. One Nepali study [1] reported 3.62 pg AFB1-lysine/mg albumin (geometric mean) in children ages 15–36 months, while two other studies in African children [32, 33] reported levels ranging 4.5–8.3 pg/mg.

The positive associations between weekly maize and groundnut consumption and serum AFB_1_-lys adduct concentrations are consistent with previous research as contamination is common in these commodities [34–37]. Maize and groundnut products have been known to commonly exceed the permissible limit for aflatoxin [34, 38–40]. While maize and groundnut production is low in the Banke area these two foods seem to be important sources of aflatoxin exposure.

Groundnuts are a nutrient-dense food, high in protein, fats, fiber, and multiple micronutrients and are a common snack in Nepal. They have recently gained popularity through government promotion programs [41]. Commercialization of groundnut products and market trends present an opportunity for spreading awareness and targeted measures to improve the quality of groundnut and groundnut products. Awareness campaigns and aflatoxin reduction interventions can help reduce consumption of aflatoxin-contaminated foods without compromising demand for nutrient-dense food items.

Aflatoxin M_1_, a hydroxylated metabolite of AFB_1_, can be found in milk or milk products from livestock that have ingested contaminated feed. Although it was beyond the scope of this study to measure AFM_1_, our study did examine the association between consuming milk and serum AFB_1_-lysine adduct concentration levels. Our findings showing positive associations between milk consumption during the past year and increased aflatoxin levels are in line with Kafle et al. [42] showing 44% of milk samples contaminated with aflatoxin M_1_. Indirect sources of contamination such as milk should not be overlooked when designing aflatoxin reduction interventions.

Although this study did not find an association between rice consumption and aflatoxin levels, rice cannot be disregarded as it is a fundamental component of the Nepali diet and can harbor low levels of aflatoxin [43]. Future work should also examine other commonly contaminated, ubiquitous foods and spices, such as black pepper, nutmeg, cumin, coriander, garlic, and dairy products (e.g., curd) [44].

Previous research suggests that DD reduces the amount of aflatoxin-prone foods consumed and counteracts adverse effects of aflatoxin [16]. Our study, with a population reliant on rice, found no association between higher DD and lower aflatoxin exposure. Findings suggest that those who diversified their diets with groundnuts or maize increased their exposure to aflatoxins. Nevertheless, DD promotion, which brings important benefits, should continue in nutrition interventions. Focused actions to lower contamination risk in these two foods should be prioritized in nutrition strategies designed to promote DD.

Seasonal variations in serum aflatoxin levels were apparent in this study, with the highest levels of exposure seen during the dry, cool winter. This strong association between AFB_1_-lysine adduct concentrations and winter season is consistent with the previous literature [2, 45–47]. Higher consumption of contaminated foods can come from either increased quantity consumed after harvest and/or consumption of lower quality, more contaminated foods that had been stored for long periods of time in either the household or market. Maize and groundnuts are typically harvested between August and September when optimum conditions for *Aspergillus* growth prevail. Prolonged, multimonth postharvest storage and suboptimal drying and storage conditions in hot, humid areas can lead to increased aflatoxin production during winter.

This study was the first to measure the association of maize and groundnut consumption and DD with maternal aflatoxin levels in pregnant women in Nepal. Findings can be used to plan interventions aimed at lowering exposure to aflatoxin, particularly in vulnerable populations. The results are generalizable because the large sample size reflected the communities represented and women were sampled from varied sociodemographic and economic circumstances. Furthermore, the outcome variable, maternal AFB_1_-lys adduct concentration, was objectively measured using HPLC. The use of QR in the analysis was an important methodological contribution not found in previous research, which has mostly relied on OLS and logistic regression. Unlike OLS, QR does not assume normality or homoscedasticity of errors and is much less influenced by extreme values of serum AFB_1_-lys. QR produced a more nuanced picture of the effects of maize and groundnut consumption patterns and DD on maternal aflatoxin exposure.

Our study has limitations. Some of the variation observed in AFB_1_-lys may be explained by factors we did not account for (e.g., quantities consumed, quality of the aflatoxin-prone foods consumed, food preparation methods, or individual variation in overall xenobiotic loads) [48]. Second, the study did not measure consumption over the previous 2–3 month period that is characteristic of AFB_1_-lys adduct half-life in the body. Finally, due to the rolling nature of the recruitment process aflatoxin data during autumn months were not available.

Results confirmed widespread aflatoxin exposure in pregnancy and showed that consumption of maize and/or groundnut consumption are dietary contributors of aflatoxin even in areas with rice-based diets. Our findings strongly support further consideration of targeted regulatory, agricultural, and food safety interventions across the value chain and at the household level to reduce aflatoxin exposure. Aflatoxin reduction campaigns should inform pregnant women and their families of both the nutritional value of consuming maize and groundnuts and of the special precautions that should be taken when purchasing, storing, and consuming agricultural food items susceptible to aflatoxin contamination. A combination of proven practical, low-cost aflatoxin reduction techniques (e.g., removal of contaminated kernels) at the household level and market level regulation of aflatoxin-prone foods could help reduce exposure to aflatoxin in vulnerable populations.
